# Artificial Intelligence in Script Concordance Test Development: A Practical Guide for Medical Educators

**DOI:** 10.1007/s40670-026-02909-y

**Published:** 2026-09-29

**Authors:** Enjy Abouzeid, Moataz Sallam

**Affiliations:** 1 School of Medicine, Ulster University, Londonderry, UK; 2 Ophthalmology Department, Suez Canal University, Ismailia, Egypt

**Keywords:** Script Concordance Test (SCT), Clinical reasoning, Artificial intelligence (AI), Generative AI, Medical education

## Abstract

Generative artificial intelligence (AI) offers new opportunities to improve the efficiency and scalability of Script Concordance Test (SCT) development in medical education. However, its use raises important concerns regarding validity, fairness, clinical accuracy and alignment with curricular outcomes. This monograph provides a practical guide for educators on using AI to design SCTs, including blueprinting, prompt construction, expert panel development, scoring, psychometric evaluation, bias auditing and sustainable item banking. AI should function as an adjunct to expert judgement, ensuring defensible, valid and educationally meaningful assessment while preserving authentic clinical reasoning.

**Supplementary Information:**

The online version contains supplementary material available at https://doi.org/10.1007/s40670-026-02909-y.

## Introduction

Generative artificial intelligence (AI) is transforming medical education assessment, offering opportunities to enhance efficiency, scalability and innovation. Script Concordance Tests (SCTs), which assess clinical reasoning under conditions of uncertainty [1, 2], have emerged as a promising application for AI-assisted assessment development. Emerging studies demonstrate the feasibility of using large language models and customised AI tools to generate SCT cases and items and to explore AI-supported approaches to expert reference-panel processes [3–6].

The potential value of generative AI in SCT development extends beyond the generation of individual test items. For experienced educators, manually constructing a single SCT item may not itself represent a substantial burden, particularly because AI-assisted drafting still requires expert verification and refinement. Its greater potential may lie in supporting the broader assessment-development workflow, including generating and adapting multiple clinical scenarios across blueprint domains and learner levels, supporting reference-panel and scoring processes, facilitating systematic quality checks and iterative refinement, and organising sustainable item banks. Accordingly, the rationale for AI integration should not be reduced to replacing faculty item writing or eliminating human workload. Rather, AI may function as a scalable adjunct within a human-governed SCT development lifecycle, with educators retaining responsibility for clinical accuracy, validity and final assessment decisions.

**Fig. 1:**
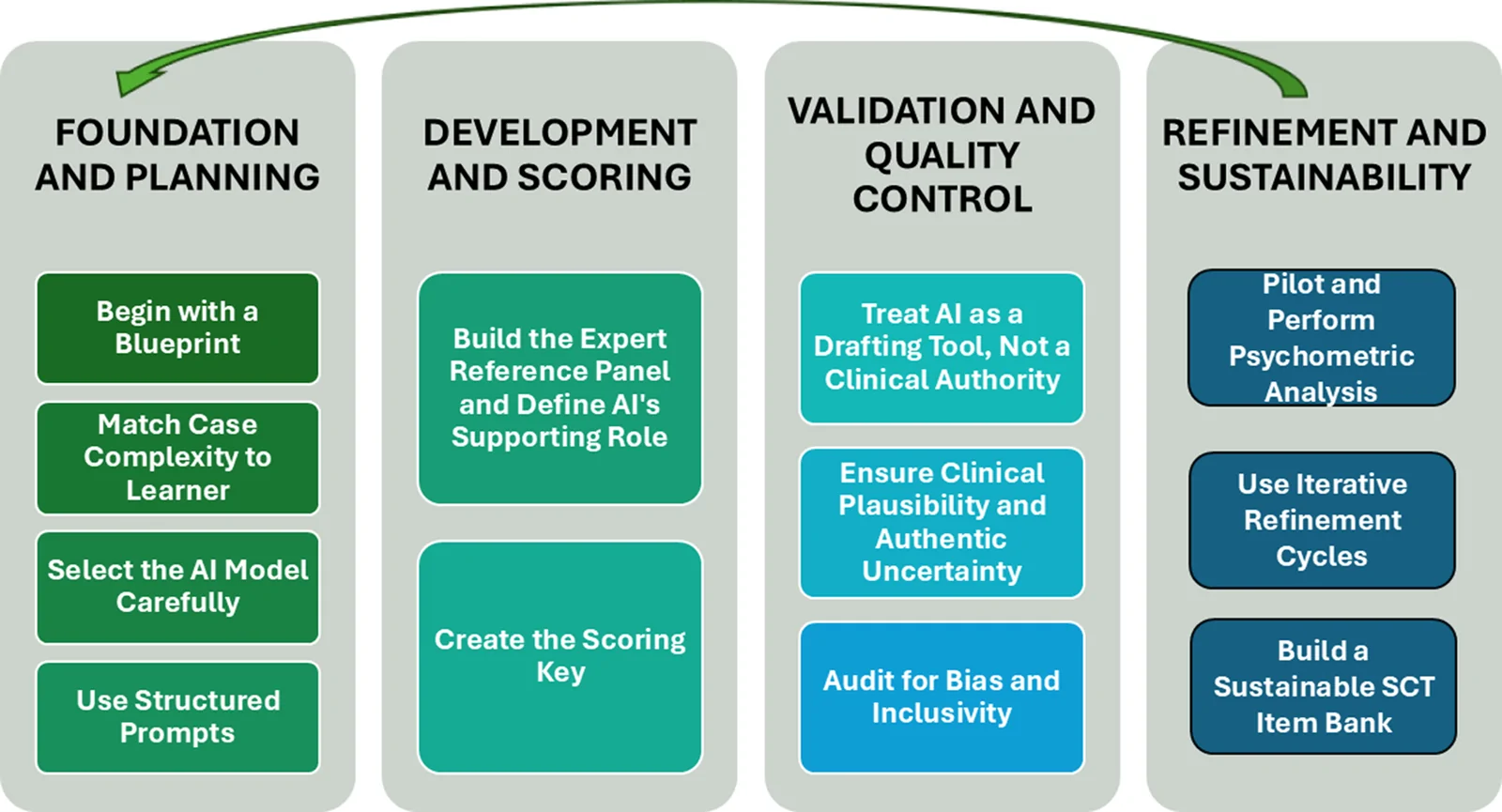
Twelve-step framework for AI-assisted Script Concordance Test development across four interconnected stages: foundation and planning, development and scoring, validation and quality control, and refinement and sustainability. The framework is iterative rather than strictly linear, with findings from validation, pilot testing and psychometric evaluation informing subsequent cycles of development and refinement

Although prompt engineering, hallucination and algorithmic bias are now well recognised in the medical education literature, guidance on AI use should move beyond describing these issues in isolation. This article therefore proposes a framework comprising 12 interconnected steps organised across four stages of the SCT development lifecycle: foundation and planning, development and scoring, validation and quality control, and refinement and sustainability, as shown in Fig. 1. The framework identifies where AI may add value, where expert judgement remains indispensable, and how AI-supported outputs should progress from blueprinting and generation through validation, psychometric evaluation, refinement and sustainable item banking.

## Step 1: Begin with a Blueprint to Preserve Content Validity

Assessment blueprinting ensures alignment between learning outcomes, instructional content and assessment items, supporting meaningful interpretation of test scores and reducing construct under-representation [7, 8]. In medical education, blueprinting is widely recognised as a key source of validity evidence and enhances fairness, transparency and defensibility in assessment design [9, 10].

As AI systems lack intrinsic curricular awareness, human-defined blueprints remain essential to preserve content validity [3]. Before generating AI-assisted SCT items, educators should explicitly follow a structured approach to ensure alignment with curricular outcomes and prevent the generation of irrelevant or redundant items. Educators should define:

- Target learner level.
- Clinical domains and conditions.
- Competency domains (diagnosis, investigation, management, prognosis).
- Cognitive complexity.

For example, when designing AI-assisted SCT items for a Year 3 respiratory block, the blueprint should first define the target learner level as students transitioning to clinically oriented reasoning. It should then specify key conditions to be sampled (e.g., pneumonia, asthma and chronic obstructive pulmonary disease) and the competency domains to be assessed, such as diagnosis, investigation, management and prognosis. Finally, it should determine the expected cognitive complexity, focusing on interpretation of clinical data and application of reasoning rather than recall.

## Step 2: Match Case Complexity to Learner Development

SCTs assess clinical reasoning in uncertain clinical contexts and are sensitive to differences in expertise across training levels [1, 2]. As learners progress, reasoning evolves from rule-based approaches to more flexible, script-based and probabilistic thinking.

When using AI to generate SCT cases, scenario complexity should align with the intended learner level, varying in diagnostic ambiguity, contextual complexity and information density. Misalignment may result in tasks that are either overly simplistic or cognitively overwhelming, reducing assessment validity. For novice learners, cases should include clear clinical cues, limited comorbidity and focus on foundational reasoning processes. In contrast, more advanced learners benefit from scenarios involving competing hypotheses, atypical presentations and incomplete or conflicting information, requiring prioritisation and nuanced decision-making, as shown in Table 1.

**Table 1:** Features of SCT cases across developmental stages of clinical reasoning

Novice learners require:	Advanced trainees benefit from:
• Clear clinical cues • Limited comorbidities • Foundational reasoning tasks • 3 Likert scale	• Competing hypotheses • Atypical presentations • Conflicting diagnostic data • 5 Likert scale

For early learners (e.g., Year 1), prompts should focus on straightforward clinical presentations and foundational reasoning processes. For example: “Generate a simple case of asthma focusing on common symptoms and first-line management.” Such cases should emphasise recognition of typical features and basic decision-making. These cases support novice learners who are still developing foundational illness scripts and rely primarily on rule-based reasoning.

For more advanced learners (e.g., Year 5), prompts should introduce greater diagnostic ambiguity and clinical complexity. For example: “Generate a complex asthma case involving comorbidities and atypical features requiring diagnostic differentiation.” These cases should require interpretation of investigation findings, prioritisation of competing diagnoses and justification of management decisions.

## Step 3: Select the AI model carefully

Multiple large language models (LLMs) are now available, and they share broadly similar capabilities in generating SCT content and following SCT formats. However, some models may generate more detailed and well-structured outputs, whereas others may produce responses that more closely approximate expert clinical reasoning or demonstrate greater internal consistency across items. For example [4], showed that different AI models varied in how closely their response patterns aligned with expert reference panel distributions, with some models demonstrating outputs that were more consistent with clinician judgement than others. While other comparative studies indicate that models such as ChatGPT and Claude are generally capable of producing plausible, contextually relevant SCT vignettes, hypotheses and response options across clinical domains [5]. These differences influence how suitable a model is for specific educational purposes, particularly when higher levels of reasoning fidelity or consistency are required.

Selecting an appropriate model should be guided by the intended educational purpose. Key considerations include the model’s ability to generate clinically plausible scenarios, follow structured SCT formats, and produce consistent outputs across multiple items. For applications requiring closer alignment with expert reasoning, such as scoring development or high-stakes assessment, models that demonstrate stronger concordance with expert responses may be preferable. In contrast, for formative use or early-stage drafting, models that prioritise fluency and efficiency may be sufficient. Practical considerations, including accessibility, customisation and workflow integration, should also inform selection.

In addition to general-purpose models, customised GPT tools offer a more targeted approach to SCT development. For example [3], demonstrated that customised GPT-based workflows, the *Script Concordance Advisor*, can efficiently generate diverse SCT items when guided by structured prompts, while still requiring careful human evaluation to ensure clinical accuracy and curricular alignment. Similarly, tools such as the *Script Concordance Test Generator*, developed by [5], illustrate how tailored AI applications can streamline SCT item development by producing structured vignettes, hypotheses and response options in the expected format.

## Step 4: Use Structured Prompts to Enhance Output Quality

The quality of AI-generated SCT items depends on the structure and specificity of the prompts used. Well-designed prompts that clearly define the task, clinical context, learner level and expected output format improve the accuracy, coherence and consistency of generated content [11–13]. In contrast, vague prompts often produce superficial, unrealistic or poorly aligned items [6].

For example, prompts that specify the required components of a clinical vignette, such as patient demographics, presenting complaint, relevant history, investigation findings and the expected type of question, can ensure that AI-generated items follow a consistent structure suitable for review and editing, as shown in Table 2. According to [3, 5], a practical approach is to use prompt templates that embed these parameters, ensuring reproducibility and reducing variability across generated items. This improves output quality and reduces the need for extensive post-generation editing.

**Table 2:** Prompt template for AI-assisted script concordance test (SCT) item generation

Prompt template
*You are a medical educator designing a Script Concordance Test (SCT) to assess clinical reasoning. Generate SCT questions based on authentic clinical scenarios that reflect decision-making under conditions of uncertainty. Each item should include a clinical vignette*,* a diagnostic or management hypothesis*,* and a new piece of information. Respondents should judge how the new information affects the hypothesis using a Likert scale.*
Key prompt components
Specialty Specify the clinical discipline (e.g., ophthalmology, cardiology, respiratory medicine). Target learner group Define the learner level (e.g., undergraduate medical students, postgraduate trainees, or continuing professional development participants). Clinical problem or topic Identify the clinical presentations or conditions to be included in the case scenarios. Focus of the questions Indicate the clinical reasoning domain being assessed (e.g., diagnosis, investigation selection, management decisions, prognosis). Guideline source Specify the reference framework for clinical decisions (e.g., NICE guidelines) or request alignment with reliable clinical guidelines and/or expert consensus. Response scale Define the SCT response scale (e.g., five-point Likert scale − 2 to + 2, or three-point scale − 1 to + 1). REQUIREMENTS: - Follow SCT format with clinical vignette, hypothesis, and new information - Maintain clinical uncertainty (avoid deterministic conclusions) - Use appropriate medical terminology - Include plausible distractors DISCLAIMER: All outputs must be verified for: ✓ Clinical accuracy against current guidelines ✓ Physiological plausibility ✓ Appropriate cognitive complexity ✓ Absence of bias or stereotyping ✓ Alignment with learning objectives

## Step 5: Establish the Expert Reference Panel and Define the Supporting Role of AI

SCT scoring reflects the degree of concordance between learners’ responses and those of an expert reference panel, capturing the variability inherent in clinical reasoning under uncertainty [1, 2]. The reliability of this approach depends on the composition and size of the panel, with evidence suggesting that approximately 15–20 experts are required to achieve stable scoring distributions [14]. However, assembling and sustaining such panels can be resource-intensive due to limited faculty availability and competing clinical demands.

Recent research has explored the potential role of artificial intelligence in supporting SCT reference panels. Although AI-generated responses may approximate plausible reasoning patterns, they do not fully replicate the contextual judgment and experiential knowledge of clinicians [4]. Accordingly, AI should not replace human experts as the primary source of scoring.

Therefore, the choice of reference panel composition should depend on the purpose of the SCT. For high-stakes assessments or complex clinical scenarios, reliance on human expert panels remains essential to ensure defensible and credible scoring. In contrast, for formative assessment, teaching activities, or learner practice, AI-assisted panels may help reduce faculty workload while still providing meaningful feedback, if educators retain oversight and validate the generated responses.

## Step 6: Create the scoring key

In practice, the most widely used approach is the aggregate scoring method, in which a panel of experienced clinicians completes the SCT beforehand, and their responses’ distribution forms the scoring key. For each answer, the credit is the number of members who chose that answer, divided by the modal value for the question. If, for a given question, 15 panel members chose “-2,” two chose “-1” and one chose “0” credit for the “-2” is 1 (15/15), credit for the “-1” is 0.13 (2/15), and credit for the “0” is 0.06 (1/15). For the non-chosen options, “+1” and “+2,” the credit is 0. With this method, all questions have the same maximum (1) and minimum (0) value. Scores obtained on each question are added to obtain a total score for the test. This number is then divided by the number of questions and multiplied by 100 to get a percentage score [15]. However, it is important to acknowledge that the optimal SCT scoring method is still debated [16].

AI can support the development and calibration of scoring keys by enhancing both efficiency and consistency. AI can support the calculation of response distributions and normalisation processes, particularly when managing large item banks or multiple response panels. It may also reduce repetitive manual processing. It may also help identify irregular or poorly discriminating items, supporting iterative refinement of the scoring framework. However, despite these advantages, AI outputs require careful review to ensure that scoring remains clinically meaningful and aligned with authentic reasoning processes. Human expert judgement, therefore, remains essential, particularly in high-stakes contexts, with AI best positioned as a supplementary tool to support efficiency and scalability.

## Step 7: Treat AI as a Drafting Tool, not a Clinical Authority

AI-generated educational content should always be reviewed critically because LLMs may produce outputs that appear highly plausible yet contain factual inaccuracies, outdated information, or overconfident conclusions [11, 17, 18]. This phenomenon, often described as hallucination, occurs because LLMs generate responses probabilistically based on patterns learned from training data rather than through genuine understanding or independent verification of facts [18, 19].

Empirical studies demonstrate that AI systems can perform well on medical knowledge assessments and licensing-style examinations, reflecting strong language fluency and the ability to integrate clinical information [20, 21]. However, performance is inconsistent, and responses may include errors in reasoning, overgeneralisation or unwarranted confidence. This variability highlights the limitations of AI as a source of authoritative clinical judgement.

Accordingly, AI should be used as a drafting tool to support item generation rather than as a substitute for expert decision-making. Human expertise remains essential to interpret, refine and validate AI-generated content, ensuring alignment with current evidence, curricular objectives and professional standards.

## Step 8: Ensure clinical plausibility while preserving authentic uncertainty

Following AI generation, SCT items must undergo targeted review to ensure both clinical plausibility and fidelity to the principles of uncertainty that underpin SCT design.

This involves systematically checking that scenarios are logically coherent; laboratory values and imaging findings are realistic; factual content and clinical reasoning are accurate; and terminology and management decisions align with accepted clinical standards. Such scrutiny is essential because even well-structured AI outputs may contain subtle inconsistencies or physiologically implausible elements that could compromise assessment credibility. For example, an AI-generated vignette reporting an oxygen saturation of 110% represents a clear physiological impossibility and must be corrected or removed.

Equally important is the preservation of authentic clinical uncertainty, which lies at the core of SCT design. SCTs assess how new information modifies the likelihood of a hypothesis rather than confirming or excluding it outright, reflecting script theory and probabilistic reasoning in clinical practice [1, 2]. AI-generated items should therefore avoid deterministic phrasing and instead allow for multiple defensible responses. For example, a normal investigation result may reduce the likelihood of a diagnosis without eliminating it entirely. For instance, a chest X-ray showing no infiltrates should reduce the likelihood of pneumonia but not eliminate it, maintaining the ambiguity that SCTs are designed to capture.

## Step 9: Audit for Bias and Representation (Inclusivity)

When using generative AI to develop assessment content, educators should systematically audit outputs for potential bias and ensure inclusive representation. AI systems may reproduce historical and societal biases embedded in training data, leading to under-representation of certain groups, reinforcement of stereotypes, or limited exposure to atypical disease presentations [22–24]. Recent work on large language models further demonstrates that these systems can exhibit systematic biases in language, reasoning patterns and representation, even when designed to appear neutral [25, 26]. In medical education, such biases can compromise fairness, reduce the authenticity of clinical scenarios and negatively influence learners’ diagnostic reasoning.

In practice, a structured bias audit should be embedded as a routine quality assurance step in AI-assisted assessment design. It should ensure balanced demographic representation, inclusion of atypical presentations and avoidance of stereotypical associations. For example, AI-generated content may disproportionately depict cardiovascular disease in male patients; revising cases to include female patients with atypical presentations of myocardial infarction enhances both realism and inclusivity as shown in Table 3.

**Table 3:** Example AI prompts across stages of AI-assisted script concordance test development (full prompts available in Supplementary Material)

Stage of SCT Development	Purpose	Prompts (Supplementary files)
Blueprinting	Align with curriculum and learner level	Generate Year 3 respiratory SCT cases covering pneumonia, asthma and COPD across diagnosis, investigation, management and prognosis
Learner alignment	Match complexity to learner level	Generate a simple asthma case for Year 1 learners and a complex asthma case for Year 5 learners with competing diagnoses
Model selection	Compare AI tools	Compare ChatGPT, Claude and customised GPT for SCT generation in cardiology and recommend the most suitable model
Structured prompting	Improve output quality	Generate SCT items with a clinical vignette, hypothesis and new information using a 5-point Likert scale
Expert panels	Support reference scoring	Simulate expert panel responses for an SCT item and estimate response distributions across Likert options
Scoring key	Create aggregate scoring	Calculate scoring credits based on expert response distribution using the aggregate scoring method
Clinical plausibility	Preserve uncertainty	Generate SCT items where new information modifies rather than confirms or excludes a diagnosis
Bias audit	Improve inclusivity	Create cardiology cases with balanced gender representation, diverse demographics and atypical disease presentations
Psychometric review	Pilot test quality	Design a pilot testing plan including reliability analysis, item discrimination and learner feedback
Iterative refinement	Improve item quality	Revise SCT items based on expert feedback, pilot testing and psychometric results
Item banking	Sustainability	Organise validated SCT items using metadata tagging, version control and annual review cycles

## Step 10: Pilot Test and Perform Psychometric Analysis

Before implementation, AI-assisted SCTs should undergo pilot testing and formal psychometric evaluation to ensure that generated items meet accepted standards of reliability, validity and educational quality [2, 27, 28]. Evidence from SCT research demonstrates that well-constructed tests can achieve acceptable reliability when aligned with learning outcomes and supported by appropriately sized expert reference panels, with reported Cronbach’s alpha values typically ranging from 0.70 to 0.85, thresholds considered acceptable for educational assessment [2, 14, 29]. Whether AI-assisted SCTs achieve psychometric performance comparable to traditionally developed assessments require further empirical evaluation. Consequently, AI-assisted items should undergo the same rigorous pilot testing and psychometric evaluation as conventionally developed items before implementation, particularly in summative assessment. However, applying these principles to AI-assisted item generation is essential, as variability in AI outputs may influence item quality and consistency.

Pilot testing enables educators to identify ambiguities, assess item performance and refine content before summative use. Key analyses that AI can assist with should include reliability estimates (e.g., Cronbach’s alpha), item-level performance metrics such as discrimination indices, and stakeholder feedback from both learners and educators to evaluate clarity, realism, and perceived fairness. Triangulating quantitative psychometric data with qualitative insights enhances the robustness of evaluation and supports iterative refinement [27, 28, 30].

## Step 11: Use Iterative Refinement Cycles

Assessment development, particularly for SCTs, is inherently iterative. Given the complexity of clinical reasoning and the variability introduced by AI-assisted item generation, a cyclical workflow is essential to ensure that items are valid, reliable and educationally meaningful.

A structured process should typically include: (1) AI drafting, (2) expert revision, (3) pilot administration, (4) psychometric analysis, and (5) item modification. Each stage contributes distinct evidence to support the overall validity argument and helps refine both item quality and scoring robustness.

Importantly, this process is not linear but cyclical: multiple rounds of refinement may be required to achieve acceptable standards. Iterative development helps reduce construct drift, ensures alignment with intended learning outcomes, and progressively improves clarity, authenticity and reliability. When systematically applied, iterative workflows enable educators to harness the efficiency of AI while maintaining rigorous educational and clinical standards.

## Step 12: Embed AI-SCT Development into a Sustainable Bank

To maximise long-term impact, AI-assisted SCT development should be embedded within a sustainable, continuously evolving item bank rather than treated as a one-off activity. Given the iterative nature of assessment design and the variability of AI-generated content, establishing structured systems for storage, tagging, review and reuse is essential to maintain quality, consistency and scalability.

A sustainable SCT bank should include well-organised metadata for each item, such as clinical domain, condition, competency (e.g., diagnosis, investigation, management), cognitive level, and level of training. This enables alignment with curricular blueprints and facilitates targeted item selection for both formative and summative assessments. Version control is also critical, allowing educators to track revisions across iterative cycles and ensure that only validated items are deployed in high-stakes settings. Embedding psychometric data (e.g., reliability contribution, discrimination indices, response distributions) within the bank further supports evidence-informed decision-making and ongoing quality assurance [28].

Artificial intelligence can play a valuable role in scaling and maintaining the item bank. AI tools can support rapid generation of new cases to fill identified gaps, suggest variations of existing items to enhance diversity, and assist in tagging and categorisation. In addition, AI can help flag outdated content, detect redundancy across items, and identify areas of over- or under-representation within the blueprint. This enables a more dynamic and responsive assessment system that evolves alongside curricular needs.

## Practical Implications for Medical Schools

The successful integration of artificial intelligence into Script Concordance Test (SCT) development requires more than access to technology; it depends on institutional readiness, faculty development and clear governance structures. Medical schools adopting AI-assisted assessment should establish formal processes that ensure educational quality while supporting sustainable implementation.

Faculty development is a critical starting point. Educators require training not only in the technical use of AI tools, such as prompt construction and output refinement, but also in understanding the limitations of large language models, including hallucinations, bias and variability in clinical reasoning outputs. Faculty should be supported to critically evaluate AI-generated content and maintain responsibility for final assessment decisions rather than relying on automated outputs.

Assessment governance is equally important. Institutions should develop clear policies regarding acceptable uses of AI in assessment design, including standards for validation, documentation of human review, and transparency in how AI contributes to item generation and scoring processes. This is particularly important in high-stakes assessments, where defensibility and accountability must remain central.

Quality assurance mechanisms should also be embedded within existing assessment systems. AI-generated SCT items should undergo the same blueprinting, peer review, psychometric testing and periodic review expected of traditionally developed assessments. Version control, audit trails and item bank management systems are essential to ensure consistency and support continuous improvement.

From a resource perspective, AI may help reduce faculty workload associated with repetitive item writing and scoring preparation, particularly in large programmes with frequent assessment cycles. However, initial investment is required for faculty training, workflow redesign and the development of robust review systems. Institutions should therefore view AI as a strategic enhancement to assessment practice rather than a short-term replacement for academic expertise.

In this context, strong educational governance requires clearly defined institutional policies for acceptable AI use, designated human accountability for assessment decisions, transparent documentation of where and how AI has contributed to item development, and established quality-assurance processes including expert review, validation, psychometric evaluation, version control and periodic review. Within such governance structures, AI-assisted SCT development can support more scalable, responsive and diverse assessment systems while preserving the rigour and professional standards expected in medical education.

## Conclusion

Artificial intelligence offers opportunities to support Script Concordance Test (SCT) development by expanding item diversity and improving efficiency at selected stages, including generating multiple item variants, supporting scoring preparation, facilitating iterative review and maintaining item banks. However, these efficiencies should not be assumed at the level of individual item writing, where the time required for prompting and expert verification may offset gains from automated generation. The value of AI therefore depends on its integration within structured processes that prioritise validity, fairness and educational rigour, with human expertise remaining central to clinical verification, preservation of authentic uncertainty and expert reference scoring.

AI should be positioned as an assistive rather than replacement tool within SCT development. As generative AI continues to evolve, future research should move beyond demonstrating its capacity to generate assessment content and examine its added value within the broader assessment workflow, including comparative efficiency, assessment quality, sustainability and educational outcomes. Such evidence will be important in determining where AI meaningfully strengthens clinical reasoning assessment and where conventional expert-led approaches remain preferable.

## Supplementary Information

Below is the link to the electronic supplementary material.

Supplementary Material 1 (DOCX 32.8 KB)

## References

[1] Charlin B, Roy L, Brailovsky C, Goulet F, van der Vleuten C. The Script Concordance test: a tool to assess the reflective clinician. Teach Learn Med. 2000 Fall;12(4):189 – 95. doi: 10.1207/S15328015TLM1204_5. PMID: 11273368. 10.1207/S15328015TLM1204_5 11273368

[2] Lubarsky S, Dory V, Duggan P, Gagnon R, Charlin B. Script concordance testing: from theory to practice: AMEE guide 75. Med Teach. 2013;35(3):184–93. Epub 2013 Jan 29. PMID: 23360487. 10.3109/0142159X.2013.760036 23360487

[3] Abouzeid E, Sallam MA. AI-assisted script concordance tests: Enhancing feasibility with customized ChatGPT. Med Teach. 2026;48(5):757–60. Epub 2025 Jul 24. PMID: 40705532. 10.1080/0142159X.2025.2533405 40705532

[4] Sallam MA, Abouzeid E. Introducing AI as members of script concordance test expert reference panel: A comparative analysis. Med Teach. 2025;47(10):1637–44. 10.1080/0142159X.2025.2473620. 40056088

[5] Kıyak YS, Emekli E. Using large language models to generate script concordance test in medical education: ChatGPT and Claude. Span J Med Educ. 2025;6(1). 10.6018/edumed.636331.

[6] Yapıcı Coşkun Z, Kıyak YS, Coşkun Ö, Budakoğlu İİ, Özdemir Ö. Large language models for generating script concordance test in obstetrics and gynecology: ChatGPT and Claude. Med Teach. 2025;47(11):1767–71. 10.1080/0142159X.2025.2497888. 40305090

[7] Schuwirth LWT, van der Vleuten CPM. Programmatic assessment: From assessment of learning to assessment for learning. Med Teach. 2011;33(6):478–85. 10.3109/0142159X.2011.565828. 21609177

[8] Kane MT. Validating the interpretations and uses of test scores. J Educ Meas. 2013;50(1):1–73. 10.1111/jedm.12000.

[9] Patil SY, Gosavi M, Bannur HB, Ratnakar A. Blueprinting in assessment: A tool to increase the validity of undergraduate written examinations in pathology. Int J Appl Basic Med Res. 2015;5(Suppl 1):S76–9. 10.4103/2229-516X.162286. PMC4552073 26380218

[10] Ismail MAA, Mat Pa MN, Mohammad JAM, Yusoff MSB. Seven steps to construct an assessment blueprint: A practical guide. Educ Med J. 2020;12(1):71–80. 10.21315/eimj2020.12.1.8.

[11] Brown TB, Mann B, Ryder N, et al. Language models are few-shot learners. Adv Neural Inf Process Syst. 2020;33:1877–901.

[12] Wei J, Wang X, Schuurmans D et al. Chain-of-thought prompting elicits reasoning in large language models. Adv Neural Inf Process Syst. 2022;35:24824–24837. https://arxiv.org/abs/2201.11903. Accessed 26 Sept 2026.

[13] Kıyak YS. Beginner-Level Tips for Medical Educators: Guidance on Selection, Prompt Engineering, and the Use of Artificial Intelligence Chatbots. Med Sci Educ. 2024;34(6):1571–6. 10.1007/s40670-024-02146-1 . PMID: 39758489; PMCID: PMC11699172. PMC11699172 39758489

[14] Gagnon R, Charlin B, Coletti M, Sauvé E, van der Vleuten C. Assessment in the context of uncertainty: How many members are needed on the panel of reference of a script concordance test? Med Educ. 2005;39(3):284–91. 10.1111/j.1365-2929.2005.02092.x. 15733164

[15] Charlin B, van der Vleuten CPM. Standardized assessment of reasoning in contexts of uncertainty: The script concordance approach. Eval Health Prof. 2004;27(3):304–19. 10.1177/0163278704267043. 15312287

[16] Bland AC, Kreiter CD, Gordon JA. The psychometric properties of five scoring methods applied to the script concordance test. Acad Med. 2005;80(4):395–9. 10.1097/00001888-200504000-00019. 15793026

[17] Bender EM, Gebru T, McMillan-Major A, Shmitchell S. On the dangers of stochastic parrots: Can language models be too big? In: *Proceedings of the 2021 ACM Conference on Fairness, Accountability, and Transparency.* 2021. pp. 610–623. 10.1145/3442188.3445922

[18] Ji Z, Lee N, Frieske R, et al. Survey of hallucination in natural language generation. ACM Comput Surv. 2023;55(12):1–38. 10.1145/3571730.

[19] Achiam J et al. GPT-4 Technical Report. arXiv:2303.08774. 2023. Available from: https://arxiv.org/abs/2303.08774

[20] Kung TH, Cheatham M, Medenilla A, et al. Performance of ChatGPT on USMLE: Potential for AI-assisted medical education using large language models. PLOS Digit Health. 2023;2(2):e0000198. 10.1371/journal.pdig.0000198. PMC9931230 36812645

[21] Gilson A, Safranek CW, Huang T, Socrates V, Chi L, Taylor RA, Chartash D. How Does ChatGPT Perform on the United States Medical Licensing Examination (USMLE)? The Implications of Large Language Models for Medical Education and Knowledge Assessment. JMIR Med Educ. 2023;9:e45312. 10.2196/45312. Erratum in: JMIR Med Educ. 2024;10:e57594. doi:10.2196/57594. PMC9947764 36753318

[22] Obermeyer Z, Powers B, Vogeli C, Mullainathan S. Dissecting racial bias in an algorithm used to manage the health of populations. Science. 2019;366(6464):447–53. 10.1126/science.aax2342 31649194

[23] Rajkomar A, Hardt M, Howell MD, Corrado G, Chin MH. Ensuring fairness in machine learning to advance health equity. Ann Intern Med. 2018;169(12):866–72. 10.7326/M18-1990 PMC6594166 30508424

[24] Azer LH, Zatarah R, Waldrip S, Ke JXC, Moukheiber M, Khanna AK, Hicklen RS, Moukheiber L, Moukheiber D, Ma H, Mathur P. Bias in artificial intelligence algorithms and recommendations for mitigation. PLOS Digit Health. 2023;2(6):e0000278. 10.1371/journal.pdig.0000278. PMC10287014 37347721

[25] Laurito W, Davis B, Grietzer P, Gavenčiak T, Böhm A, Kulveit J. AI–AI bias: Large language models favor communications generated by large language models. Proc Natl Acad Sci U S A. 2025;122(31):e2415697122. 10.1073/pnas.2415697122. PMC12337326 40729390

[26] Navigli R, Conia S, Ross B. Biases in Large Language Models: Origins, Inventory and Discussion. J Data Inf Qual. 2023;15(2):1–21. 10.1145/3597307. Article 10.

[27] Downing SM. Validity: on meaningful interpretation of assessment data. Med Educ. 2003;37(9):830-7. 10.1046/j.1365-2923.2003.01594.x. PMID: 14506816. 14506816

[28] Norcini J, Anderson B, Bollela V, Burch V, Costa MJ, Duvivier R, Galbraith R, Hays R, Kent A, Perrott V, Roberts T. Criteria for good assessment: consensus statement and recommendations from the Ottawa 2010 Conference. Med Teach. 2011;33(3):206 – 14. doi: 10.3109/0142159X.2011.551559. PMID: 21345060. 10.3109/0142159X.2011.551559 21345060

[29] Abouzeid E, Sallam MA. Teaching by concordance: Individual versus team-based performance. Innov Educ Teach Int. 2023;60(4):513–23. 10.1080/14703297.2022.2061568.

[30] Tavakol M, Dennick R. Making sense of Cronbach’s alpha. Int J Med Educ. 2011;2:53–5. 10.5116/ijme.4dfb.8dfd . PMID: 28029643; PMCID: PMC4205511. PMC4205511 28029643

