# Supplementary material for: Artificial Intelligence in Script Concordance Test Development: A Practical Guide for Medical Educators
Source: Med Sci Educ. 2026 Sep 29;36(6):2765–72. doi: 10.1007/s40670-026-02909-y (PMC13759877; doi:10.1007/s40670-026-02909-y)
Supplement: Supplementary file 1 [file 40670_2026_2909_MOESM1_ESM.docx]

**Prompts for Using AI in Script Concordance Tests_ Supplementary files**

**Tip 1:** **Begin with a Blueprint to Preserve Content Validity**

***Prompt:***

"You are designing a Script Concordance Test for Year 3 medical students on respiratory medicine. Create a blueprint that specifies:

- Target learner level: Year 3 undergraduate medical students transitioning to clinical reasoning

- Clinical domains: Pneumonia, asthma, COPD

- Competency domains: Diagnosis (recognizing presentations), Investigation (selecting tests), Management (first-line treatments), Prognosis (disease progression)

- Cognitive complexity: Application and analysis rather than recall

Generate 3 SCT cases that align with this blueprint, ensuring representative sampling across all domains."

**Tip 2** **Match Case Complexity to Learner Development**

***Prompt for novice learners (Year 1-2):***

"Generate a simple asthma case for Year 1 medical students focusing on:

- Clear clinical cues: Classic wheeze, cough, shortness of breath

- Limited comorbidities: Otherwise healthy patient

- Foundational reasoning: Identify symptoms and basic pathophysiology

- Response scale: 3-point Likert (-1 to +1)

Include straightforward history, typical triggers, and first-line bronchodilator treatment."

***Prompt for advanced learners (Year5+):***

"Generate a complex asthma case for Year 5 students featuring:

- Competing hypotheses: Asthma vs COPD vs heart failure vs anxiety

- Atypical presentations: Incomplete response to treatment, overlapping features

- Conflicting diagnostic data: Mixed investigation results

- Comorbidities: Obesity, GERD, or cardiovascular disease

- Response scale: 5-point Likert (-2 to +2)

- Require diagnostic differentiation and management prioritization under uncertainty."

**Tip 3: Select the AI model carefully**

***Prompt:***

"Compare the following AI models for generating Script Concordance Test items in cardiology:

1. ChatGPT-4

2. Claude 3

3. Customized GPT (Script Concordance Advisor)

For each model, evaluate:

- Ability to follow SCT format structure

- Clinical accuracy of generated content

- Consistency with expert reasoning patterns

- Handling of clinical uncertainty

- Quality of distractors and response options

- Provide a comparative analysis and recommend the most suitable model for high-stakes assessment versus formative learning purposes."

**Tip 4** **Use Structured Prompts to Enhance Output Quality**

**Prompt template**

You are a medical educator designing a Script Concordance Test (SCT) to assess clinical reasoning. Generate SCT questions based on authentic clinical scenarios that reflect decision-making under conditions of uncertainty. Each item should include a clinical vignette, a diagnostic or management hypothesis, and a new piece of information. Respondents should judge how the new information affects the hypothesis using a Likert scale.

**Key prompt components**

**Specialty**
Specify the clinical discipline (e.g., ophthalmology, cardiology, respiratory medicine).

**Target learner group**

Define the learner level (e.g., undergraduate medical students, postgraduate trainees, or continuing professional development participants).

**Clinical problem or topic**

Identify the clinical presentations or conditions to be included in the case scenarios.

**Focus of the questions**

Indicate the clinical reasoning domain being assessed (e.g., diagnosis, investigation selection, management decisions, prognosis).

**Guideline source**

Specify the reference framework for clinical decisions (e.g., NICE guidelines) or request alignment with reliable clinical guidelines and/or expert consensus.

**Response scale**

Define the SCT response scale (e.g., five-point Likert scale −2 to +2, or three-point scale −1 to +1).

**Tip 5:** **Build diverse expert panels; experts and AI models**

***Prompt***

"You are assisting in developing a hybrid expert panel for SCT scoring.

TASK: Generate preliminary scoring distributions for the following SCT item:

[Insert SCT vignette]

INSTRUCTIONS:

1. Simulate responses from a panel of 15-20 expert clinicians

2. For each response option (-2 to +2), estimate the percentage of experts who would select it

3. Identify which options are most/least defensible

4. Flag any response patterns that suggest item ambiguity

NOTE: This AI-generated distribution will be compared against actual human expert panel responses for validation. Human expert judgment remains essential for high-stakes assessment scoring."

**Tip 6: Create the scoring key**

***Prompt***

"You are calculating an SCT scoring key using the aggregate scoring method.

INPUT: Expert panel responses for Question 1:

- Option -2: 12 experts

- Option -1: 3 experts

- Option 0: 2 experts

- Option +1: 0 experts

- Option +2: 0 experts

TASK:

1. Calculate credit for each option (responses/maximum responses)

2. Normalize scores to 0-1 scale

3. Identify the modal response

4. Flag any irregular patterns (e.g., bimodal distributions, low discrimination)

5. Provide scoring key in format suitable for LMS import

Show all calculations and explain the clinical reasoning behind the response distribution."

**Tip 7 Treat AI as a Drafting Tool, Not a Clinical Authority**

***Prompt***

"You are generating DRAFT SCT items for expert review.

IMPORTANT: These items require critical appraisal by medical educators before use.

TASK: Generate 5 draft SCT cases on [topic] for [learner level]

REQUIREMENTS:

- Follow SCT format with clinical vignette, hypothesis, and new information

- Maintain clinical uncertainty (avoid deterministic conclusions)

- Use appropriate medical terminology

- Include plausible distractors

DISCLAIMER: All outputs must be verified for:

✓ Clinical accuracy against current guidelines

✓ Physiological plausibility

✓ Appropriate cognitive complexity

✓ Absence of bias or stereotyping

✓ Alignment with learning objectives

Human expert review is mandatory before any educational use."

**Tip 8: Ensure clinical plausibility while preserving authentic uncertainty**

***Prompt***

"You are generating an SCT case that preserves authentic clinical uncertainty.

CLINICAL SCENARIO: Chest pain in emergency department

REQUIREMENTS:

1. Create a vignette where new information MODIFIES (not confirms/excludes) the hypothesis

2. Avoid physiological impossibilities (e.g., O2 sat >100%, negative heart rate)

3. Ensure laboratory values are realistic and internally consistent

4. Maintain ambiguity: Multiple defensible interpretations should exist

5. Use probabilistic language: "reduces likelihood" not "rules out"

EXAMPLE OF POOR PHRASING: "Normal troponin rules out MI"

EXAMPLE OF GOOD PHRASING: "Normal troponin reduces the likelihood of acute MI but does not exclude it entirely, especially if presentation was early"

Generate 3 items that exemplify these principles."

**Tip 9 Audit for Bias and Representation (Inclusivity)**

***Prompt***

"You are generating SCT cases with attention to bias and inclusive representation.

TASK: Create 5 cardiology cases that ensure:

DEMOGRAPHIC DIVERSITY:

- Gender balance: Include both male and female patients

- Age range: Young adults to elderly

- Ethnic diversity: Reflect varied populations

- Socioeconomic contexts: Different backgrounds

CLINICAL REPRESENTATION:

- Atypical presentations (e.g., women with MI presenting without classic chest pain)

- Avoid stereotypical associations (e.g., not all diabetics are obese)

- Include less common disease presentations

LANGUAGE AUDIT:

- Culturally sensitive terminology

- No stigmatizing language

- Person-first language where appropriate

After generating cases, perform a bias audit checklist:

✓ Gender distribution balanced?

✓ Diverse patient demographics?

✓ Atypical presentations included?

✓ No stereotypical associations?

✓ Culturally appropriate language?"

**Tip 10: Pilot Test and Perform Psychometric Analysis**

***Prompt***

"You are designing a pilot test for AI-generated SCT items.

ASSESSMENT: 20-item SCT on respiratory medicine for Year 4 students

PILOT PLAN:

1. Sample size: Minimum 30 students for psychometric analysis

2. Data collection:

- Item-level statistics: Difficulty, discrimination index

- Reliability: Cronbach's alpha (target 0.70-0.85)

- Response distribution patterns

- Time to completion

3. Qualitative feedback:

- Student survey: Clarity, realism, fairness (5-point Likert)

- Educator review: Clinical accuracy, appropriateness

- Open-ended comments on problematic items

4. Success criteria:

- α ≥ 0.70

- Item discrimination > 0.20

- No items with >20% ambiguous responses

- Positive stakeholder feedback

Generate a pilot test report template and analysis plan."

**Tip 11: Use Iterative Refinement Cycles**

***Prompt***

"You are managing an iterative refinement cycle for SCT item development.

CURRENT STATUS: Round 2 of refinement

ITEM: [Insert SCT case]

PREVIOUS FEEDBACK:

- Round 1 expert review: "Wording unclear in stem, revise investigation values"

- Round 1 pilot: Discrimination index = 0.15 (below threshold)

- Student feedback: "Confusing new information"

REFINEMENT TASKS:

1. Revise clinical vignette for clarity

2. Adjust laboratory values to be more realistic

3. Simplify language while maintaining complexity

4. Ensure new information clearly modifies (not confirms) hypothesis

5. Re-calculate scoring key based on revised format

NEXT STEPS:

- Round 3 expert review (n=5)

- Round 3 pilot testing (n=30)

- Compare psychometric metrics across rounds

- Document all changes for quality assurance

Track this item through 3-5 cycles until acceptable reliability achieved."

**Tip 12: Embed AI-SCT Development into Sustainable Bank**

***Prompt***

"You are managing a sustainable AI-enhanced SCT item bank.

ITEM BANK SPECIFICATIONS:

METADATA TAGGING SCHEMA:

- Clinical domain: [Cardiology/Respiratory/GI/etc.]

- Condition: [Specific diagnosis]

- Competency: [Diagnosis/Investigation/Management/Prognosis]

- Cognitive level: [Remember/Understand/Apply/Analyze]

- Learner level: [Year 1-5/Resident/Fellow]

- Difficulty: [Easy/Medium/Hard based on pilot data]

- Psychometric data: [Discrimination index, reliability contribution]

- Version: [v1.0, v2.0, etc.]

- Last review date: [Date]

- Status: [Draft/Under review/Validated/Retired]

QUALITY ASSURANCE WORKFLOW:

1. AI generation → 2. Expert review → 3. Pilot testing → 4. Psychometric analysis → 5. Validation → 6. Deployment → 7. Annual review

AI-ASSISTED FUNCTIONS:

- Identify gaps in blueprint coverage

- Flag outdated content (>3 years old)

- Detect redundant items

- Suggest variations for existing cases

- Auto-tag new items based on content analysis

- Generate reports on bank composition

GOVERNANCE:

- Quarterly expert review cycles

- Annual content updates aligned with guidelines

- Retirement criteria: Poor discrimination, outdated evidence

- Version control for all modifications

Generate a comprehensive item bank management plan."
